# Solution Blowing Spinning Technology towards Green Development of Urea Sensor Nanofibers Immobilized with Hydrazone Probe

**DOI:** 10.3390/polym13040531

**Published:** 2021-02-11

**Authors:** Mohamed H. El-Newehy, Hany El-Hamshary, Waheed M. Salem

**Affiliations:** 1 Department of Chemistry, College of Science, King Saud University, Riyadh 11451, Saudi Arabia; helhamshary@ksu.edu.sa; 2 Department of Chemistry, Faculty of Science, Tanta University, Tanta 31527, Egypt; 3 Technology of Medical Laboratories Department, Menoufia University, Shebin-El Koum 32513, Egypt; waheedsalem1979@gmail.com

**Keywords:** solution blowing spinning, cellulose nanofibers, hydrazone probe, urease, urea biosensor

## Abstract

Cellulose has been one of the most widespread materials due to its renewability, excellent mechanical properties, biodegradability, high absorption ability, biocompatibility and cheapness. Novel, simple and green colorimetric fibrous film sensor was developed by immobilization of urease enzyme (U) and tricyanofuran hydrazone (TCFH) molecular probe onto cellulose nanofibers (CNF). Cellulose acetate nanofibers (CANF) were firstly prepared from cellulose acetate using the simple, green and low cost solution blowing spinning technology. The produced CANF was exposed to deacetylation to introduce CNF, which was then treated with a mixture of TCFH and urease enzyme to introduce CNF-TCFH-U nanofibrous biosensor. CNF were reinforced with tricyanofuran hyrazone molecular probe and urease enzyme was encapsulated into calcium alginate biopolymer to establish a biocomposite film. This CNF-TCFH-U naked-eye sensor can be applied as a disposable urea detector. CNF demonstrated a large surface area and was utilized as a carrier for TCFH, which is the spectroscopic probe and urease is a catalyst. The biochromic CNF-TCFH-U nanofibrous biosensor responds to an aqueous medium of urea *via* a visible color signal changing from off-white to dark pink. The morphology of the generated CNF and CNF-TCFH-U nanofibrous films were characterized by different analytical tools, including energy-dispersive X-ray patterns (EDX), polarizing optical microscope (POM), Fourier-transform infrared spectroscopy (FT-IR) and scanning electron microscope (SEM). SEM images of CNF-TCFH-U nanofibers demonstrated diameters between 800 nm and 2.5 μm forming a nonwoven fabric with a homogeneous distribution of TCFH/urease-containing calcium alginate nanoparticles on the surface of CNF. The morphology of the cross-linked calcium alginate nanoparticles was also explored using transmission electron microscopy (TEM) to indicate an average diameter of 56–66 nm. The photophysical performance of the prepared CNF-TCFH-U was also studied by CIE Lab coloration parameters. The nanofibrous film biosensor displayed a relatively rapid response time (5–10 min) and a limit of detection as low as 200 ppm and as high as 1400 ppm. Tricyanofuran hydrazone is a pH-responsive disperse dye comprising a hydrazone detection group. Determination of urea occurs through a proton transfer from the hydrazone group to the generated ammonia from the reaction of urea with urease.

## 1. Introduction

Chronic kidney failure has been a common global syndrome that may result in death due to a significant increase in the discharging degree of body metabolism wastes to blood stream. For instance, urea [CO(NH_2_)_2_] is generally released by kidney into blood and urine as a body metabolic waste [1,2,3]. However, the concentration level of blood urea in a chronic kidney failure patient is usually in the range of 50–70 mM, which could increase up to 10 folds its value in normal individuals (2.5–7.1 mM). Therefore, the amount of urea in both blood and urine can be an indicative tool for patients at different stages of kidney failure [4,5,6]. There have been various methods reported for both qualitative and quantitative detection of urea, such as gas chromatography isotope dilution mass spectrometry and electrochemical tools [7,8]. Nonetheless, those methods are complicated, requires highly trained personnel, low sensitive, costly and time consuming. Therefore, there has been increasing demands for the preparation of simple and cost-effective diagnostic devices for clinical applications. The typical design of these diagnostic devices usually utilizes nanomaterials comprising spectroscopic active sites with the ability to recognize the presence of a particular analyte by colorimetric and/or fluorescence methods [9,10]. However, fluorescent sensors usually suffer paramagnetic fluorescence quenching, especially in biological fluids after binding with metallic ions. Moreover, the application of fluorescent sensors in aqueous solutions is restricted due to their low solubility. They also necessitate complicated sensor assembly and costly purification [11,12,13]. Thus, the development of a colorimetric sensor consisting of a hosting nanofibrous film mat and active spectroscopic sites is more promising. Such colorimetric diagnostic tool is characterized by higher sensitivity and specificity, portability, low cost, simplicity of preparation and real-time visual determination of the targeted analyte. In addition, it needs less labor and simple instrumentations [14,15,16,17,18]. Nanomaterials have been significant for a diversity of applications, such as drug delivery, ecological monitoring, foodstuffs processing and medical diagnostic tools [19,20,21,22]. Although, there have been a broad range of well-known nanomaterials amended to establish detection capability, cellulose nanofibers (CNF) have established a low attention in this regard. CNF exhibit a number of positive characteristics, such as renewability, biodegradability, biocompatibility, low density, low cost, abundance, as well as large and amendable surface area [21,23,24]. Additionally, CNF have been applied largely to reinforce various polymer composites. In this context, CNF demonstrated a great potential in a variety of engineering applications, such as molecular biology, food packaging, filtration membranes, paper making, sensors and biosensors, pharmaceutics, optical and electronic devices, coating additives and cosmetics [25,26,27,28,29]. Electrospinning has been used to produce polymer-based nanofibers. Nonetheless, electrospun nanofibers are generally costly and produced in a very low yield. In addition, electrospinning is restricted to viscoelastic materials and the homogeneous porosity cannot be controlled [30,31,32]. Electrospinning has been used to produce electrospun CNF starting from electrospinning of cellulose acetate (CA) followed by deacetylation [33]. This could be ascribed to the very low electroconductivity of cellulose in comparison to the higher electroconductivity of cellulose acetate [34,35,36,37]. On the other side, the recently innovated solution blowing spinning is characterized with simplicity, lower cost, higher production rate and safer as it does not require high voltage as in the case of electrospinning [38,39].

Biosensors have been applied in a variety of applications, such as foodstuffs, medical and pharmaceutical purposes, as well as environmental monitoring [40]. Compared to other analytical methods, enzyme-based diagnostic biosensors are characterized with higher sensitivity and specificity as well as lower cost [41]. Both trypsin and α-chymotrypsin enzymes have been immobilized by adsorption onto a microcrystalline cellulose. Nonetheless, such technique suffers from reversibility and therefore it is not scalable [42,43]. As a main difficulty in developing an effective enzymatic biosensor, it must be assured that the immobilized enzymatic substrate can be held reactive over time [44]. The incorporation of urease enzyme enclosed within a light-activated polymer can afford an efficient sensor for the detection of CO(NH_2_)_2_ for a broad range of concentrations (0.5–15 mM) with fast response time (1–2 min) [9,45]. Enzymatic substrates can be linked to CNF *via* physical, covalent, and salt-bridge bond formation. The properties of CNF can present a biocompatible medium for the enzyme detection to occur [46]. Glucose oxidase, attached to a nanocomposite of gold nanoparticles and cellulose nanowhiskers *via* thiol bonds of polyaziridine, was capable to act as a naked-eye sensor introducing different extents of activities relying on the amount of thiol cross-linker [47]. Urease covalently attached to cellulose fibers and activated by glutaraldehyde was found to noticeably improve the biosensor stability against temperature [48]. The concurrent incorporation of several constituents in a preparation procedure of a biosensor tool has been a challenging process in the development of an inclusive detection device. Thus, the co-immobilization of those constituents into an inactive polymer matrix, usually polyacrylamide, has been utilized *via* micro-emulsion polymerization [49]. This method is efficient as optical sensor. However, it is a difficult process to co-encapsulate the enzyme in combination with a peptide as organic solvents that are usually used in the encapsulation course. Alginates do not have such drawback because it has been utilized in aqueous environments under gentle circumstances. The active layer enclosing both a probe and an enzyme can be formed *via* alginate cross-linking by replacing Na^+^ with Ca^2+^ ions [50].

Herein, we developed urea biosensor *via* the co-immobilization of urease enzyme and TCFH probe onto CNF. Calcium alginate acted as nanofiller with the ability to attach TCFH probe and urease enzyme into CNF mat, which applied as chromogenic nanofibrous mat for real-time colorimetric detection of aqueous urea. This urea biosensor is useful for on-site determination of urea. TCFH chromophore comprising a tricyanofuran (TCF) acceptor heterocycle and a hydrazone (H) donor moiety was generated *via* simple azo-coupling of TCF and diazonium salt of 2,4-dinitrophenylamine. TCFH spectroscopic sensors have been used as multi-stimuli responsive molecular switcher in a variety of sensing applications, such as halochromic, biochromic, vapochromic and solvatochromic purposes. They have been used as sensors for the detection of toxic gases such as ammonia, pH indicators monitoring acid/base conditions and sensors for bacteria. This could be attributed to their excellent characteristics, such as thermal and photochemical stability [9]. The cross-linked calcium alginate [(COO-)_2_ Ca^2+^] fixes both TCFH probe and urease enzyme by forming an outer layer on CNF-TCFH-U nanofibrous film. The high porosity and large surface area of CNF present a higher adsorption on the nanofibrous surface of the miniaturized CNF as well as an improved diffusion within the nanofibrous sensor bulk. Thus, the prepared CNF-TCFH-U nanofibrous biosensor demonstrated an improved sensitivity upon exposure to NH_3_ discharged from reacting CO(NH_2_)_2_ with urease. The colorimetric performance, reversibility and limit of detection were investigated. Different analysis methods, including EDX, POM, TEM, FT-IR and SEM, were utilized to inspect the properties of the formed nanofibrous architectures.

## 2. Materials and Methods

### 2.1. Materials and Reagents

Urease enzyme from Canavalia ensiformis (Jack bean), sodium alginate, cellulose acetate (Mn 30,000; acetyl content of ~39.8 wt%) and calcium chloride were supplied from Sigma-Aldrich (St Louis, MO, USA). Triple distilled water was employed in the current study. All solvents and materials were supplied from commercial supplier and were utilized as received. TCFH chromophore was prepared as described by Khattab, et al. [9]. Tricyanofuran (TCF) was synthesized from propanedinitrile and 3-hydroxy-3-methylbutan-2-one (2:1 molar ratio) in the presence of a sodium ethoxide solution in absolute ethanol to give an off-white crystalline solid with a yield of 58%; mp 201–203 °C; ^1^HNMR (400 MHz, CDCl_3_): δ 2.37 ppm (s, 3H), δ 1.64 ppm (s, 6H). TCFH was prepared *via* azo-coupling of 2,4-dinitrophenylamine diazonium chloride with TCF to offer an orange-red powder in moderate yield of 68%; mp 234–236 °C; ^1^HNMR (400 MHz, DMSO-*d_6_*): δ 12.14 ppm (s-broad, 1H), δ 8.61 ppm (s, 1H), δ 8.40 ppm (d, 1H), δ 8.26 ppm (d, 1H), δ 7.68 ppm (d, 1H), δ 1.84 ppm (s, 6H); IR (neat): 3253 cm^−1^ (secondary amine), 2223 cm^−1^ (cyano), 1577 cm^−1^ (C=N), 1507 cm^−1^ and 1322 cm^−1^ (nitro). The synthetic reactions were observed by TLC Merck aluminum plates PF-254. To ensure high purity, both TCF intermediate and TCFH chromophore were subjected to purification by re-crystallization.

### 2.2. Preparation of Cellulose Nanofibers (CNF)

The solution blowing spinning consists of spinning nozzle, air compressor, fiber collector and injections pump. A viscous solution of cellulose acetate (15 wt%) in acetone was stirred for 2 h at room temperature. The generated solution was pumped through a 19-gauge needle at a bar atmospheric pressure of 5 × 10^−1^ and a flowing rate of 10 mL h^−1^. The fiber collector was placed at a distance of 45 cm from the spin nozzle. The spin needle was placed in the middle of concentric nozzle, whilst the protruding was carried out at a 1 mm out of the concentric nozzle. The generated cellulose acetate nanofibers were subjected to deacetylation process in an aqueous medium of sodium hydroxide (0.1 M) over 48 h to regenerate CNF, which was then rinsed with distilled water to neutralization (pH = 7) and finally air-dried under ambient conditions [27,28,29,30].

### 2.3. Preparation of Colorimetric Cellulose Nanofibers (CNF-TCFH) Sensor

**Figure 1:**
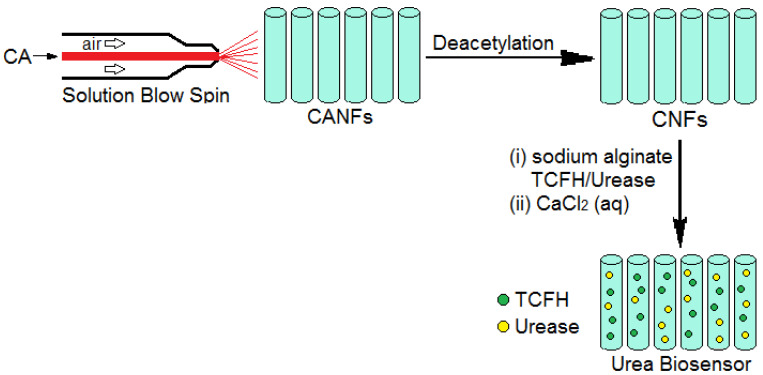
Schematic diagram displaying the preparation of cellulose nanofibrous biosensor for the detection of urea; TCFH = tricyanofuran hydrazone, CANFs = cellulose acetate nanofibers, and CNFs = cellulose nanofibers.

As shown in Figure 1, an aqueous solution of sodium alginate (0.1 g) in triple distilled water (10 mL) was mechanically stirred for 1 h under ambient conditions. Different concentrations of TCFH (0.05, 0.1, 0.5, 1.0, 1.5 and 2.0 wt% relative to the weight of the nonwoven mat of cellulose nanofibers (CNF); represented by CNF-TCFH-1, CNF-TCFH-2, CNF-TCFH-3, CNF-TCFH-4, CNF-TCFH-5 and CNF-TCFH-6) was dissolved separately in lowest amount of acetone. Each solution of TCFH in acetone was added separately to the above prepared aqueous solutions of sodium alginate (10 mL). The solutions were mechanically stirred for 1 h under ambient conditions. The produced solutions were subjected to homogenization at 8000 rpm for 5 min using a rotor-stator SRH 60-70 (Samro homogenizer Co., LTD, Shanghai, China). Urease (5 mg/mL) was added to the produced solutions, and then was stirred for 30 min. The fabricated cellulose nanofibers were subjected to padding in the prepared aqueous solutions of alginate/TCFH/urease for 5 min, left to air-dry with total moisture of 50%. CNF nonwoven mats were subjected to padding in an aqueous solution of calcium chloride (1.0 × 10^−4^ M) for 5 min. The dyed nonwoven mats (CNF-TCFH) were left to air-dry for 1 h.

### 2.4. Methods

The melting points (°C) of the synthesized TCFH chromophore were recorded uncorrected using an electrothermal equipment. NMR spectra were determined under ambient conditions by Bruker Avance 400 (Billerica, MA, USA) at 400 MHz as the chemical shift is measured in ppm. Fourier-transform infrared (FT-IR) spectroscopy of both TCFH and CNF-TCFH-U were determined in the range of 400–4000 cm^−1^ using Nexus 670 (Nicolet, Waltham, MA, USA). The morphology of CNF-TCFH-U nonwoven mats were examined with Quanta FEG250 (Prague, Czech Republic) scanning electron microscopy (SEM) at 30 kV. The average diameter of CNF and alginate nanoparticles were measured using Image J software connected to SEM. The energy-dispersive X-ray analyzer (TEAM) coupled to SEM was applied to record the chemical composition of CNF-TCFH-U nonwoven mats. EDX spectra were reported at a current of 1 nA and an acceleration voltage of 20 kV. The polarized optical microscopic (POM) images were reported on DM 750-P (Leica Ltd., Wetzlar, Germany) attached to a reflection kit as well as Link Sys 32 program to capture data. Both morphology and particle size of the formed calcium alginate nanoparticles were explored by transmission electron microscope (TEM; acceleration voltage of 100 kV) JEOL-1230 (JEOL, Tokyo, Japan). The nanofibrous CNF-TCFH-4 sample was rinsed with distilled water and centrifuged for 30 min. The nanofibrous nonwoven mat was removed away from the produced aqueous dispersion, which was homogenized for 20 min. Then, a drop of the produced aqueous dispersion containing calcium alginate nanoparticles was poured onto a copper grid. The colorimetric measurements of CNF-TCFH-U nonwoven mats, including UV-visible absorption spectra, CIE Lab (a*, b* and L*) as well as colorimetric strength (K/S), were tested by UltraScan® PRO (HunterLab, Reston, VA, USA). Where, L* is lightness from blackest (0) to whitest (100) levels, a* is the range of colors extending from reddest (+) to greenest (−) levels, and b* is the range of colors extending from yellowest (+) to bluest (−) levels. The pH value was monitored by ADWA digital AD-11 pH meter.

### 2.5. Urea Sensing

The CNF-TCFH biosensor was tested at pH = 6.8 employing a buffer solution of phosphate/EDTA (1.0 × 10^−1^ M) under ambient conditions. The determination of urea was investigated at different concentrations (1–1700 ppm). The off-white CNF-TCFH sensor was soaked in every urea solution for 5–10 min at 37 °C to introduce a dark pink color as a result of the generation of ammonia after hydrolysis process among urease and CO(NH_2_)_2_. Photos of CNF-TCFH-U previous to and following exposure to an aqueous solution of urea were recorded by Canon A710 IS digital camera (Tokyo, Japan).

## 3. Results and Discussion

### 3.1. Preparation of Cellulose Nanofibers Biosensor for Urea

**Scheme 1:**
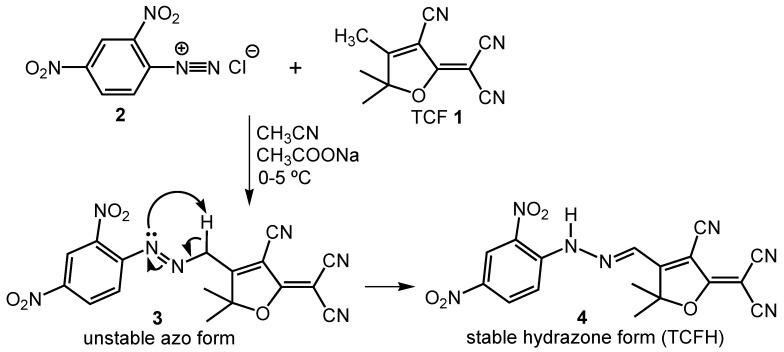
Synthesis of TCFH spectroscopic probe 4.

The solution blowing spinning technology was applied on a viscous solution of cellulose acetate in acetone to generate cellulose acetate nanofibrous (CANF) mat, which was then deacetylated in an aqueous medium of sodium hydroxide to produce cellulose nanofibers [34]. An aqueous mixture of sodium alginate, urease enzyme and TCFH at different concentrations in triple distilled water was applied onto the prepared CNF nonwoven mats *via* the pad-dry-cure technique. The dyed nonwoven mats (CNF-TCFH) were then padded in an aqueous solution of calcium chloride to generate cross-linked nanoparticles of calcium alginate [(COO-)_2_ Ca^2+^] enclosing both urease enzyme and TCFH spectroscopic active sites. The inspiration of this research intended to fabricate cellulose-based nanofibrous biosensor with large surface area and high porosity for colorimetric determination of urea. TCFH was synthesized in a moderate yield of 68 % from the azo-coupling of TCF 1 heterocycle with diazonium salt of 2,4-dinitrophenylamine 2 in a weakly basic (sodium acetate) solution in acetonitrile. This weak alkali is capable for the deprotonation of the reactive CH_3_ substituent on the strongly electron withdrawing tricyanofuran heterocycle. The azo-coupling reaction generates an unstable azo-containing entity 3, which rearranges to the stable hydrazone-containing TCFH 4 (Scheme 1).

### 3.2. Morphological Characterization

**Figure 2:**
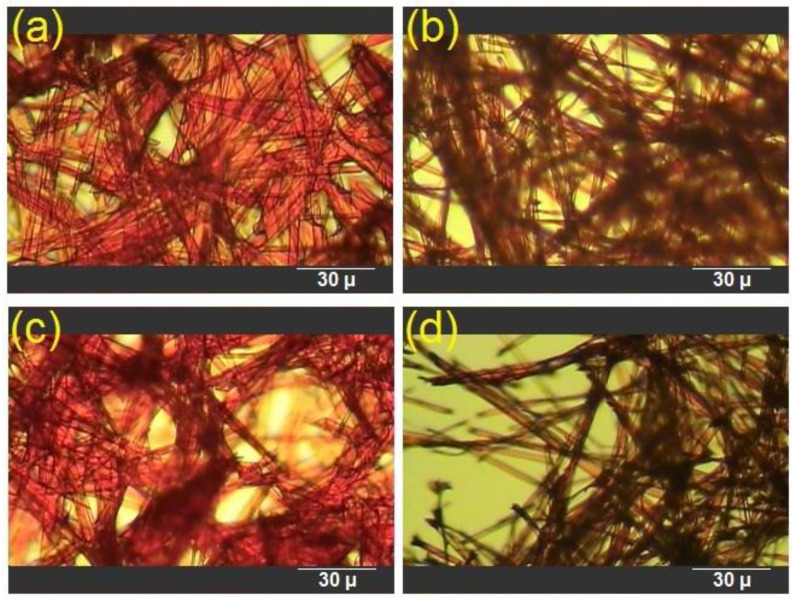
Polarizing optical microscope images of the generated nanofibers before (cellulose acetate nanofibers; (**a**,**b**)) and after deacetylation (cellulose nanofibers; (**c**,**d**)).

**Figure 3:**
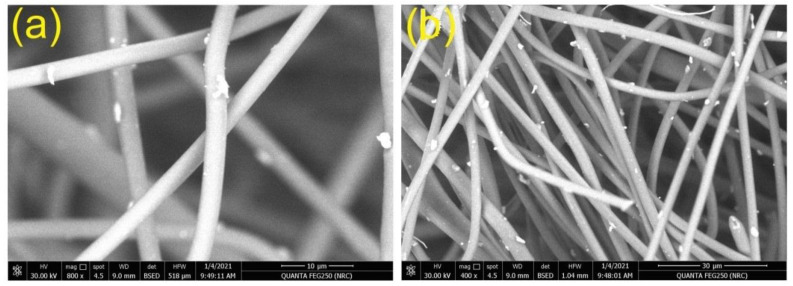
SEM images of CNF-TCFH-1 (**a**) and CNF-TCFH-6 (**b**) nonwoven mats.

Nanomaterials have been renowned with their excellent surface functionalization, high porosity and large surface area. Thus, cellulose nanofibers colorimetric biosensor can be reported as a significant solid detection tool for the onsite sensing of a trace targeted analyte. In comparison to solution-based colorimetric sensors, solid sensors have been typically portable, simple, easy to prepare, inexpensive, fast and practical, which make them better candidates for basic laboratory assays for commercial and household applications. Therefore, the search for an eco-friendly solid-state biosensor has been of great significance. The three-dimensional films prepared from CNF demonstrate high interconnectivity, large surface area and good porous bulk. These distinguished properties may lead to potential applications of highly efficient and miniaturized solid-state biosensors. The polarizing optical microscope (POM) was firstly utilized to confirm the production of both CANF and CNF from the solution blowing spinning technology. POM images confirmed no differences took place to the morphological properties of the generated nanofibrous mats before (CANF) and after (CNF) deacetylation as displayed in Figure 2. The structural morphologies of the prepared CNF-TCFH nonwovens were investigated by SEM images and EDX results as displayed in Figure 3 and Table 1, respectively. The nanofibrous nonwoven mats showed homogeneous interconnectivity of a network with large surface area and high porosity. In comparison to blank CNF nanofibrous network, CNF-TCFH-U samples showed nanostructures of calcium alginate layer enclosing TCFH probe and urease enzyme deposited onto CNF. The produced calcium alginate nanoparticles displayed a consistent distribution on the surface of CNF. This could be ascribed to the improved chemical cross-linking of calcium alginate to the surface of CNF. The diameters of the produced CNF were between 800 nm and 2.5 μm, whereas the diameters of the calcium alginate nanoparticles were in the range of 56–66 nm. These miniaturized nanostructures are preferred to enhance the sensing ability of urea because they offer a higher surface area leading to a higher adsorption of urea analyte onto the nanofibrous surface as well as a better porosity leading to an improved diffusion within the nanofibrous sensor bulk. Therefore, the prepared CNF-TCFH-U biosensor exhibited an improved sensitivity upon exposure to NH_3_ liberated from reacting urease enzyme with CO(NH_2_)_2_ [51]. The morphologies of CNF-TCFH-U nanofibers showed increasing in the density of calcium alginate nanoparticles with increasing their concentration.

**Figure 4:**
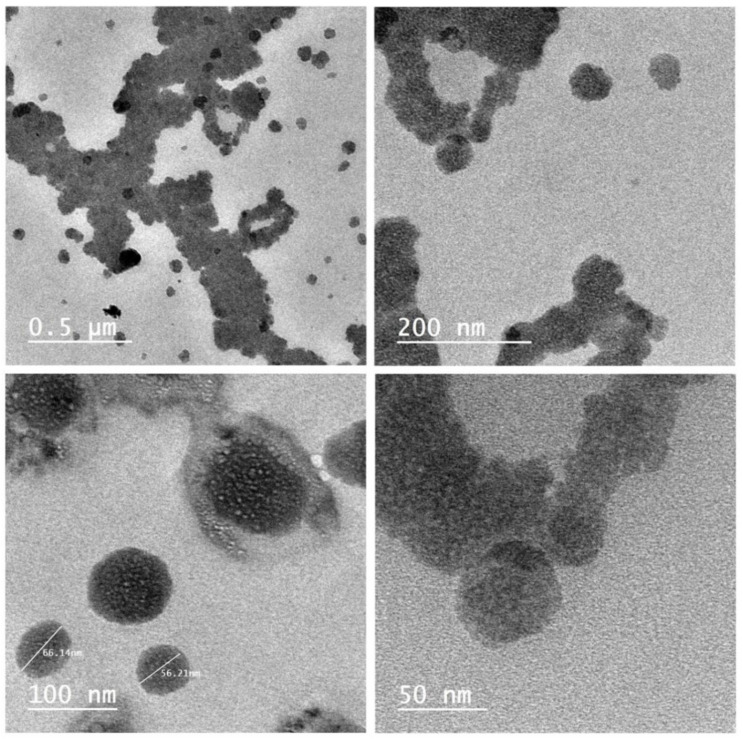
TEM images of calcium alginate nanoparticles isolated from CNF-TCFH-4.

The elemental compositions of CNF-TCFH samples were also reported by energy dispersive X-ray (EDX) spectroscopy by scanning two different spots on the surface of the inspected CNF-TCFH samples as depicted in Table 1. The elemental compositions of the monitored elements were approximately the same at the selected two scanned spots to prove a homogeneous distribution of the calcium alginate nanoparticles into the surface of CNF. The calcium alginate nanoparticles morphology and their size were investigated by TEM images (Figure 4) to designate an average size of 56–66 nm. To create an aqueous suspension of calcium alginate nanoparticles immobilized onto CNF, CNF-TCFH-4 was subjected to rinsing and centrifuging.

**Figure 5:**
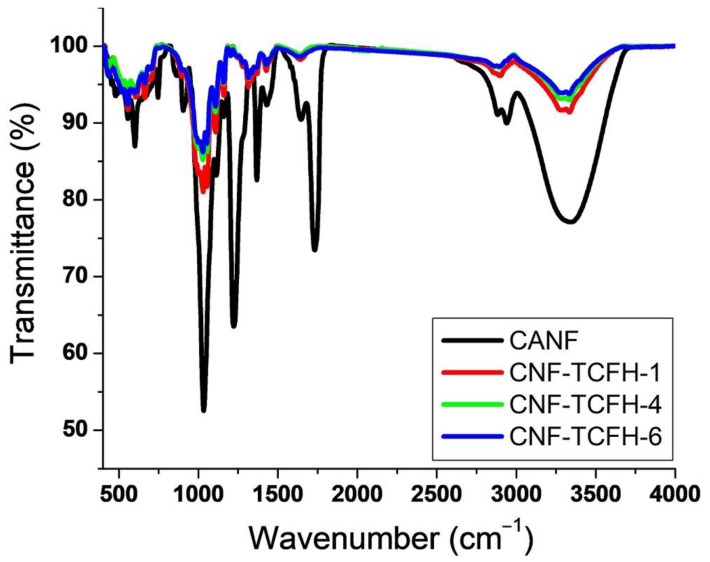
FT-IR spectra of CANF, CNF-TCFH-1, CNF-TCFH-4 and CNF-TCFH-6.

FT-IR spectra were utilized to confirm the successful deacetylation of CANF into CNF. FT-IR spectrum of CANF displayed distinctive bands attributed to the acetate stretching vibrations at 1228 cm^−1^ owing to ether group, 1369 cm^−1^ owing to C–CH_3_ group, and 1739 cm^−1^ owing to carbonyl group. Due to deacetylation, the characteristic peaks demonstrated in the spectrum of CNF were ascribed to the decreased intensity of the hydroxyl (OH) which shifted from 3328 cm^−1^ for CANF to 3339 cm^−1^ for CNF. The characteristic acetate peaks disappeared to prove the development of CNF. FT-IR spectra of CANF, CNF-TCFH-1, CNF-TCFH-4 and CNF-TCFH-6 are shown in Figure 5. In general, no considerable differences were monitored in the absorption peaks for CNF-TCFH-1, CNF-TCFH-4 and CNF-TCFH-6. The characteristic bands of blank CNF were monitored at 3339 cm^−1^ owing to stretching hydroxyl, 2894 cm^−1^ due to stretching CH, and 1316 cm^−1^ due to wagging CH. An absorbance band was monitored at 1642 cm^−1^ owing to vibrational deformation of the adsorbed water molecules. Compared to FT-IR spectrum of blank CNF, CNF-TCFH-1, CNF-TCFH-4 and CNF-TCFH-6 didn’t display new peaks proving that no considerable chemical interactions occurred to the surface of CNF due to deposition of TCFH/urease-encapsulated in calcium alginate into CNF.

### 3.3. Activity of CNF-TCFH Biosensor

**Figure 6:**
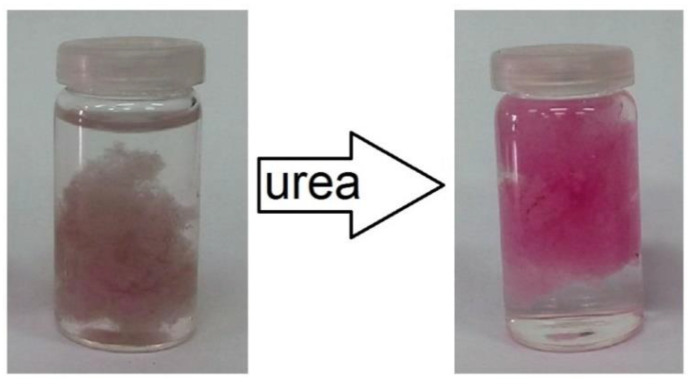
Biochromic color change due to releasing ammonia from reaction of urease with urea on the surface of CNF-TCFH-4.

The color change of the prepared biosensor was visually detected to shift from off-white to dark pink (Figure 6). The biosensor chromic activity was explored by studying the colorimetric changes in CIE (L*, a*, b*) coordinates, colorimetric strength (K/S) and absorbance spectra. The total content of TCFH in the nanofibrous CNF-TCFH composite as an active spectroscopic sites incorporated on the surface of CNF played a significant role in the biosensor chromic activity. The colorimetric screening and sensor effectiveness at different concentrations of TCFH are depicted in Table 2. Before and after immersing the nanofibrous CNF-TCFH biosensor into an aqueous solution of urea for 5–10 min, the UV-Vis absorbance spectra were recorded. After immersing of the nanofibrous CNF-TCFH biosensor into an aqueous solution of urea, the color strength was found to slightly increase to indicate a better tinctorial strength. Considerable increments were detected in K/S with increasing the total content of TCFH from CNF-TCFH-1 to CNF-TCFH-4. On the other hand, slight increments were detected in K/S with increasing the total content of TCFH from CNF-TCFH-4 to CNF-TCFH-6. This could be due to that CNF reaches equilibrium with the dye-uptake of TCFH at CNF-TCFH-4. Thus, the colorimetric screening results showed that the optimum coloration measurements were detected at CNF-TCFH-4. Exposure to an aqueous solution of urea leads to an increase in the absorbance wavelength of CNF-TCFH from 476 to 548 nm. The coloration screening CIE Lab (L*, a* and b*) parameters of the nanofibrous CNF-TCFH biosensor at different total contents of TCFH were determined previous to and following exposure to an aqueous solution of CO(NH_2_)_2_. The pristine CNF exhibited a white shade with low a* (−1.32), low b* (1.61) and high L* (89.42). The nanofibrous CNF-TCFH biosensor displayed color change from off-white to dark pink within 5–10 min, during which the urease/urea reaction generate aqueous NH^4+^ ions to increase the pH value of the urea aqueous medium. In comparison to blank CNF, all CNF-TCFH samples displayed considerably different values of CIE Lab relying on the total contents of TCFH. L* was considerably decreased to demonstrate a darker shade with increasing the total contents of TCFH from CNF-TCFH-1 to CNF-TCFH-4, which could be ascribed to the increased dye-uptake of TCFH. On the other hand, L* showed a negligible increase with increasing the total contents of TCFH from CNF-TCFH-4 to CNF-TCFH-6 due to the decreased dye-uptake of TCFH upon reaching equilibrium. L* demonstrated a considerable increase after exposure to an aqueous solution of urea as monitored by the darker shade generated on CNF-TCFH. Both a* and b* exhibited positive values, which change from white for uncoated CNF to off-white for TCFH-coated CNF-TCFH. The nanofibrous CNF-TCFH samples displayed a darker white color upon increasing the total contents of TCFH. Before exposure to urea, b* exhibited positive values, while a* demonstrated positive values, which can be translated to an off-white color. After exposure to urea, b* demonstrated negative values, while a* exhibited positive values, which can be translated to the generation of a dark pink color.

**Figure 7:**
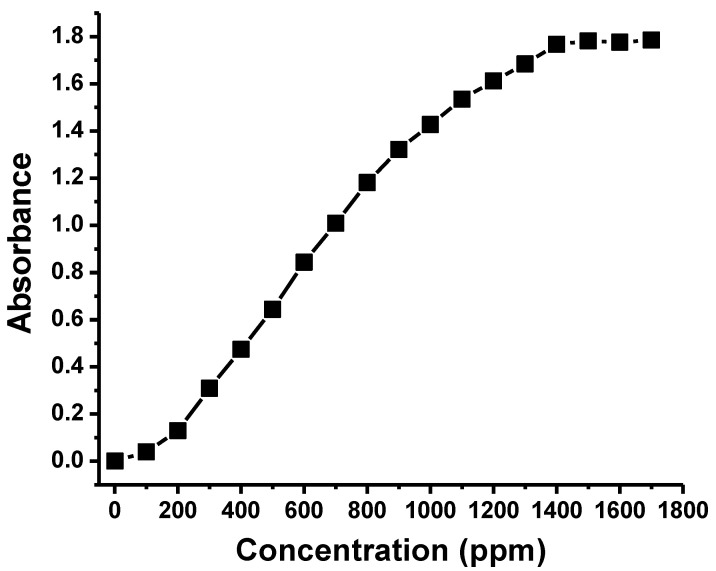
Absorbance values of CNF-TCFH-4 at 548 nm with increasing urea concentration in aqueous solution from 1 to 1700 ppm.

The nanofibrous CNF-TCFH-4 biosensor was effectively applied to estimate an unknown amount of urea in an aqueous solution relying on a calibration curve assembled for the range of concentrations between 1 and 1700 ppm as depicted in Figure 7. Upon exposing to urea, the absorbance intensity of CNF-TCFH-4 at 548 nm showed a linear curve with increasing concentration of urea in the range of concentrations between 200 and 1400 ppm. Naked-eye color change was monitored with increasing the concentration of urea due to the generated NH_3_, which subtract a proton from N-H group. The detection limit of CNF-TCFH-4 was monitored by recording its absorption at 548 nm with increasing the concentration of urea to identify the first absorption intensity value at ~0.13, which is assigned to the concentration of urea at 200 ppm. After increasing concentration of urea to 1400 ppm, no further increase in the absorbance intensity (~1.77) was detected. The absorption wavelength of CNF-TCFH-4 was also found to change from 476 nm in the absence of urea to 548 nm in the presence of urea. The nanofibrous CNF-TCFH-4 biosensor was maintained stable in a fridge for ~5 days. Figure 7 displays a calibration curve for the determination of urea using CNF-TCFH-4.

### 3.4. Mechanism of Detection

**Figure 8:**
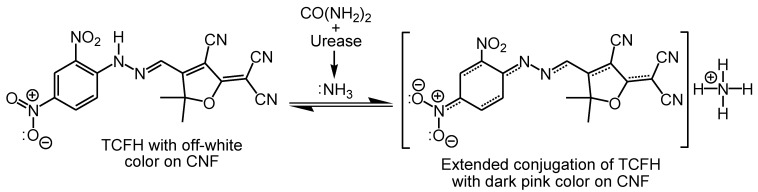
Suggested mechanism of TCFH for colorimetric recognition of urea.

The halochromism of TCFH was established by including nitro-substituted phenylhydrazone moiety acting as a strong electron-donating group after deprotonating the hydrazone N-H group to produce hydrazone anion. The electron-withdrawing 2,4-dinitro groups on the phenylhydrazone introduced a highly acidic hydrazone N-H group [52,53]. Such highly electron-withdrawing nitro substituents led to an enhancement in the stability of the produced negatively charged hydrazone anion, which in turn result in the formation of a push-pull structure with an extended conjugated molecular system. Thus, different colors were generated relying on the level of conjugation (Figure 8). The enzymatic catalytic hydrolysis reaction of urea molecules to the alkaline ammonium ions can be motivated by urease enzyme as a catalyst on the surface of the nanofibrous CNF-TCFH biosensor. Increasing the concentration of urea results in increasing the concentration of the generated ammonium ions, and consequently increasing the pH value of the aqueous solution. This in turn results in color change from off-white to dark pink. The produced positively charged ammonium cations cannot be bonded to the negatively charged carboxyl anions on calcium alginates because those carboxyl anions are already cross-linked [(COO-)_2_ Ca^2+^] to each other *via* calcium cations. Therefore, the urease-motivated hydrolysis of CO(NH_2_)_2_ to ammonium cations can be transformed to a naked-eye color change due to the deprotonation of TCFH spectroscopic chromophore by the produced alkaline ammonium ions.

## 4. Conclusions

Simple, cost-effective and green approach was developed for the production of nanofibrous cellulose nonwoven biosensor for the detection of urea. Both morphology and elemental analyses of the generated nonwoven biosensor were studied by different analytical methods, including POM, SEM, EDX and FT-IR. SEM images displayed calcium alginate nanoparticles immobilized onto the surface of the nonwoven cellulose nanofibers. SEM images of CNF-TCFH demonstrated fibrous diameters between 800 nm and 2.5 μm, while TEM images of cross-linked calcium alginate nanoparticles indicated an average diameter of 56–66 nm. Both urease enzyme and tricyanofuran hydrazone spectroscopic active cites were encapsulated into calcium alginate nanoparticles. We prepared a pH-responsive tricyanofuran hydrazone spectroscopic chromophore composed of a push hydrazone-π conjugated-pull tricyanofuran structure, which exhibited a hydrazone group acting as an electron donor moiety due to deprotonation. Increasing the concentration of the tricyanofuran hydrazone spectroscopic chromophore did not affect on the morphology of the nanofibrous biosensor; however, it influenced the colorimetric properties. The best colorimetric measurements were monitored upon using a concentration of the tricyanofuran hydrazone spectroscopic chromophore at 1.0 wt%. Combination of tricyanofuran hydrazone, urease enzyme, alginate biomolecules and cellulose nanofibers introduced a composite film comprising different colorimetric measurements relying on the concentration of the spectroscopic probe. Urease acted as a catalyst with urea analyte to generate ammonia in the presence of pH stimuli-responsive TCFH active sites to allow the determination of urea. The current technique can be applied to detect urea in biofluids, such as blood and urine. The coloration results proved that the nanofibrous cellulose detector demonstrated a detection limit of 200–1400 ppm with a good and real-time sensitivity between 5 and 10 min. Exposure to urea dissolved in aqueous medium results in a notable naked-eye color shift from off-white to dark pink. This straightforward method will pave the way to functional textiles for healthcare purposes, such as protective clothing and urea-containing foodstuffs products.

## Figures and Tables

**Table 1:** Elemental content of blank CNF and CNF-TCFH-4 at two selected spots A and B on the sample surface.

CNFs		C	O	Ca	Cl
CNF	A	55.72	44.28	0	0
	B	55.41	44.59	0	0
CNF-TCFH-4	A	52.15	46.11	1.23	0.51
	B	52.32	46.31	0.84	0.53

**Table 2:** Colorimetric screening measurements of the nanofibrous CNF-TCFH biosensor at different total contents of TCFH previous to (A) and following (B) exposure to an aqueous solution of urea (conc. 1400 ppm).

Sample Code	L		a*		b*		*K/S*	
	A	B	A	B	A	B	A	B
CNF-TCFH-1	74.12	61.20	4.19	5.54	10.83	−4.08	3.43	3.91
CNF-TCFH-2	71.73	57.03	6.28	8.34	14.78	−5.10	5.27	5.88
CNF-TCFH-3	64.19	44.45	9.22	10.27	21.61	−8.74	7.18	7.68
CNF-TCFH-4	63.28	44.91	9.38	13.66	21.49	−9.78	8.85	9.33
CNF-TCFH-5	62.88	43.20	10.78	14.55	26.37	−11.19	9.26	9.70
CNF-TCFH-6	61.49	42.45	11.24	14.97	27.62	−12.65	9.79	10.06

## Data Availability

The data presented in this study are available on request from the corresponding author.
